# Mechanical properties of rock cutting by disc cutters and feasibility analysis of mechanical mining in Jinchuan No. 3 Mine with hard rock

**DOI:** 10.1038/s41598-025-32995-8

**Published:** 2025-12-19

**Authors:** Jian Zhao, Dan Huang, Daming Zhu

**Affiliations:** 1 Institute of Mining Engineering, BGRIMM Technology Group, Beijing, 102628 PR China; 2 No.3 Mine, Jinchuan Group Co., Ltd., Gansu, 737100 PR China

**Keywords:** Hard rock breaking, Disc cutter, Mechanical mining, Wheel-type cutter head, Numerical simulation, Energy science and technology, Engineering, Solid Earth sciences

## Abstract

Disc cutters are essential rock-cutting tools for Tunnel Boring Machines (TBMs), primarily used in hard rock excavation. However, TBMs are not suitable for mining actives due to limitations in size and flexibility. This study first investigates the mechanical properties of rock cutting by disc cutters, and then explores the feasibility of mechanical mining with disc cutter based hard rock mining machines. The ores in the Jinchuan No. 3 Mine represent to typical hard rockmass, with rock strength ranging from about 125 to 180 MPa. The mechanical properties of hard rock cutting are investigated by linear cutting machine (LCM) tests and numerical simulations, showing good consistency between experimental and simulation results. The laboratory results reveal maximum normal forces of 40 kN and 60 kN at penetration depths of 2 mm and 4 mm, respectively, with specific energy (SE) of 7.33 kWh/m^3^ and 8.42 kWh/m^3^. The simulation results indicate maximum normal forces of 40 kN and 55 kN. Moreover, simulated crack propagation during rock cutting supports the tensile crack-dominant model, with crack characteristics closely matching existing theoretical analysis and experimental observations. During rock cutting, multiple semi-conical shaped cracks are generated, with their sizes initially decreasing before stabilizing. Simulation of rock cutting with double disc cutters identifies the optimal cutter spacing. Accordingly, a cutter arrangement approach for the wheel-type cutter head of hard rock mining machines is proposed. Finally, the feasibility of mechanical mining in the Jinchuan No. 3 Mine is discussed through estimating mining efficiency and proposing an innovative mining method.

## Introduction

Drilling and blasting mining methods have been widely used in both metal and non-metal mines^1–3^, but blasting operations inherently pose safety risks due to explosions and are accompanied by flying rocks and shock waves^4^, threatening workers’ safety. With the advancement of non-blasting mechanical mining technologies and machines, many countries are transitioning towards non-blasting mining methods, including China^5^, US^6^, Poland^7,8^, etc. Compared to the drilling and blasting mining method, the non-blasting mechanical mining is of less disturbance to surrounding rock and better excavation quality. It is safer and more economical, because it eliminates explosion accidents and reduces the consumption of materials such as explosives, detonators, drill rods and drill bits. What’s more, it is also a prerequisite and foundation for the future development of unmanned and intelligent mining. Non-blasting mechanical mining or excavation is common in coal mines and tunnel construction, where the former focuses on rock breaking equipment using conical picks, such as cantilever roadheaders and shearer loaders^9,10^, while disc cutter-based TBMs (tunnel boring machines) are used in the latter. Due to limited rock-breaking capacity, conical pick equipment is mainly suitable for breaking intact rock with a UCS (uniaxial compressive strength) of less than 80 MPa^11^. Of course, under the influence of rockmass joint development^12–14^, the rock-breaking capacity of the conical pick equipment could be enhanced. However, for breaking intact hard rock with UCS over 100 MPa, the cutting equipment using disc cutters is the preferred and mature choice.

In terms of the rock cutting mechanism of disc cutters, two factors contribute to rock breaking^15^: (1) cutter penetration and rolling effects on the rock to produce grooves, and (2) interaction between neighboring grooves, causing larger rock chips. From a macroscopic perspective, three reaction (cutting) force components are generally defined between the disc cutter and the rock during penetration and rolling^16^, normal force, rolling force and side force. At the microscopic level, two rock theoretical model analyze rock breaking by disc cutters: tensile crack-dominant model and shear crack-dominant model. The tensile crack-dominant model assumes that tensile failure, rather than shear failure, is the primary chip-forming mechanism of disc cutters^17^, and it is used to derive predictive equations for the cutting process. Sanio^17^ further explained that the disc cutter first produces a crushed zone under penetration, which then causes tangential tensile stresses in the undamaged rock zone. When these stresses exceed the tensile strength, tensile cracks emerge and propagate radially from the cutting edge. Conversely, the shear crack-dominant model attributes the cutting groove formation to shearing failure^18^. Paul^19^ documented that rock behaves plastically under penetration by a rigid wedge, and the force-penetration relationship follows the Mohr -Coulomb yield criterion. Miller M H^20^ also agreed that the rock failure surface is controlled by shear stress.

To better understand the rock cutting mechanism of disc cutters and optimize cutting performance, numerous scholars have studied the mechanical properties of rock cutting using single or double cutters through LCM (linear cutting machine) experiments and numerical simulations. Cho^21^ applied photogrammetric measurement to assess the excavated rock volume by a full-scale TBM disc cutter in granitic rock in Korea and performed three-dimensional numerical analysis using AUTODYN-3D to simulate rock cutting behavior. The results correlated well with LCM test data, including rolling force and specific energy. Furthermore, numerical simulations were employed to optimize the spacing of TBM disc cutters at a penetration depth of 4 mm, showing good agreement with LCM test results^22^. However, these studies neglected the crack propagation behavior and did not compare normal force between tests and simulations, which is a key parameter for determining the trust force of TBMs. Entacher^23^ investigated crack propagation beneath disc cutters and the corresponding rock failure mechanisms. Through capturing high-resolution images of crack networks, they obtained the cross-sections of typical crack patterns during LCM tests. Cracks were classified into lateral cracks and median cracks, however, the crack patterns along the rolling direction were not investigated. In the field of full-scale linear cutting tests, Thyagarajan and Rostami^24^ developed a modified testing method to achieve the LCM test under variable penetration mode, generating a continuously increasing and decreasing force-penetration plot. They found that the measured forces using the variable penetration method are slightly lower than those in conventional constant penetration tests and declared the normal force as the dominant force for rock cutting by disc cutters. Jeong^25^ analyzed the correlation between rock brittleness and cutting performance parameters with LCM and CAI (Cerchar Abrasivity Index) test results, presenting several empirical equations for predicting cutter forces, specific energy and CAI. Apart from the LCM test, numerical simulations are also widely employed to study the mechanical properties of the disc cutter cutting process, such as in mixed-face ground^26^, on composite rocks^27^, under coupled static-dynamic loading^28^, using a new disc cutter with surface spiral grooves^29^, a pre-grooving-assisted disc cutter^30^ or a disc cutter with diverse edge shapes^31^.

**Fig. 1:**
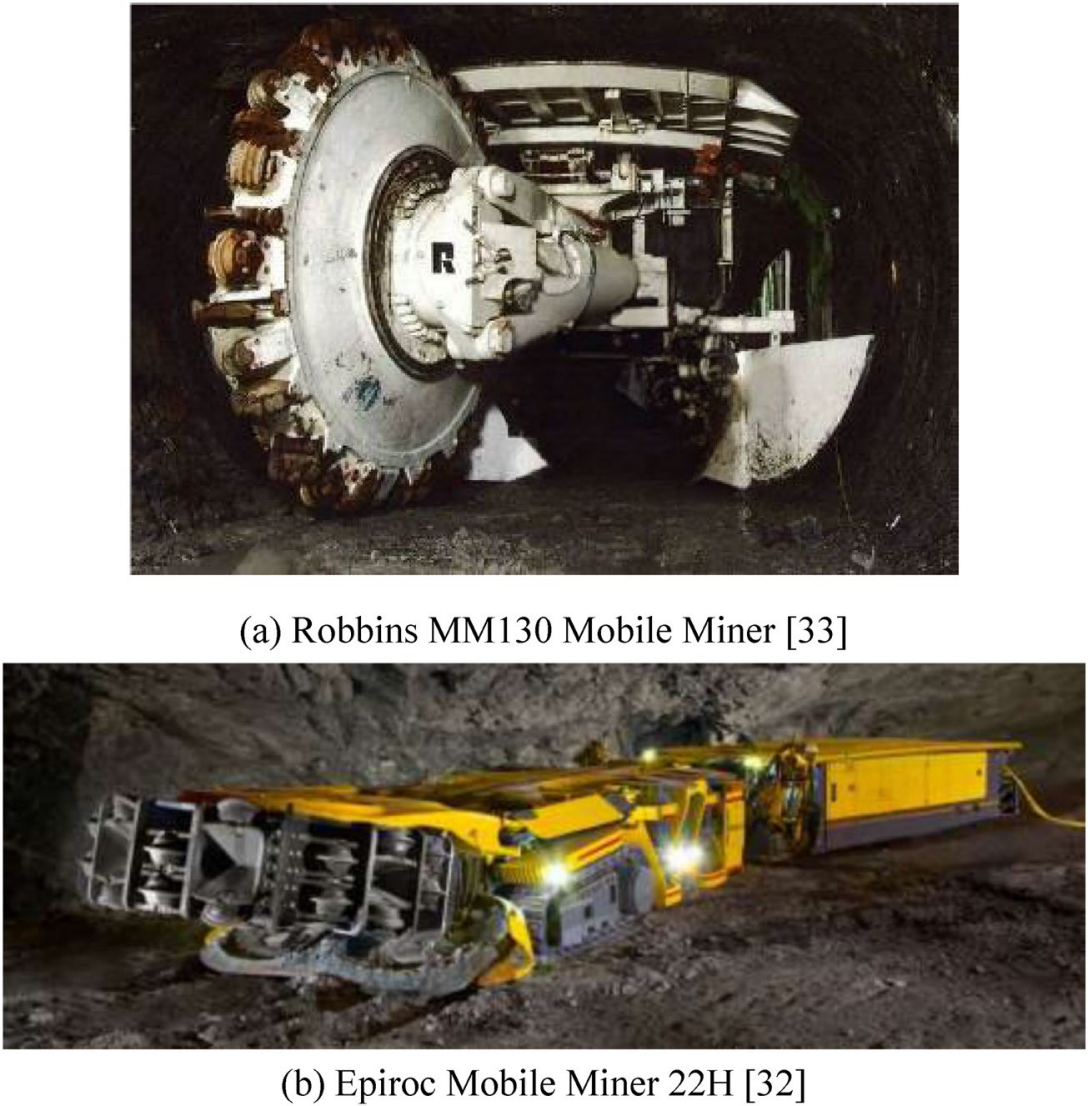
Mining equipment for breaking hard rock via disc cutters.

In addition to research on the rock cutting mechanism, advances in hard rock mining machines with disc cutters (see Fig. 1), such as the Epiroc Mobile Miner series^32^ and Robbins Mobile Miners^33^, have gradually made non-blasting mechanical mining is gradually becoming possible in hard rock mines (UCS≥100 MPa^34^). These mining machines break hard rock through a vertically or horizontally placed cutting wheel embedded numerous disc cutters on its surface. However, studies on cutter arrangements and application feasibility in geotechnical engineering constructions involving hard rocks, especially in metal and non-metal mines, remain scarce.

Consequently, this paper first studies the mechanical properties of hard rock cutting by disc cutters through full-scale LCM tests, taking the Jinchuan No. 3 Mine as an engineering case study. Then, it sets up a DEM-based numerical model of rock cutting to investigate cutting forces, crack propagation patterns and optimal cutter spacing. To ensure the reliability of the numerical simulation results, the model and its parameters are calibrated by comparing cutting forces obtained from both experimental and numerical methods. Finally, the paper puts forward an approach to designing cutter arrangements of the cutting wheel, and preliminary evaluates the feasibility of non-blasting mechanical mining using hard rock mining machines with disc cutters.

## Experimental investigation

### Rock conventional mechanical property

**Fig. 2:**
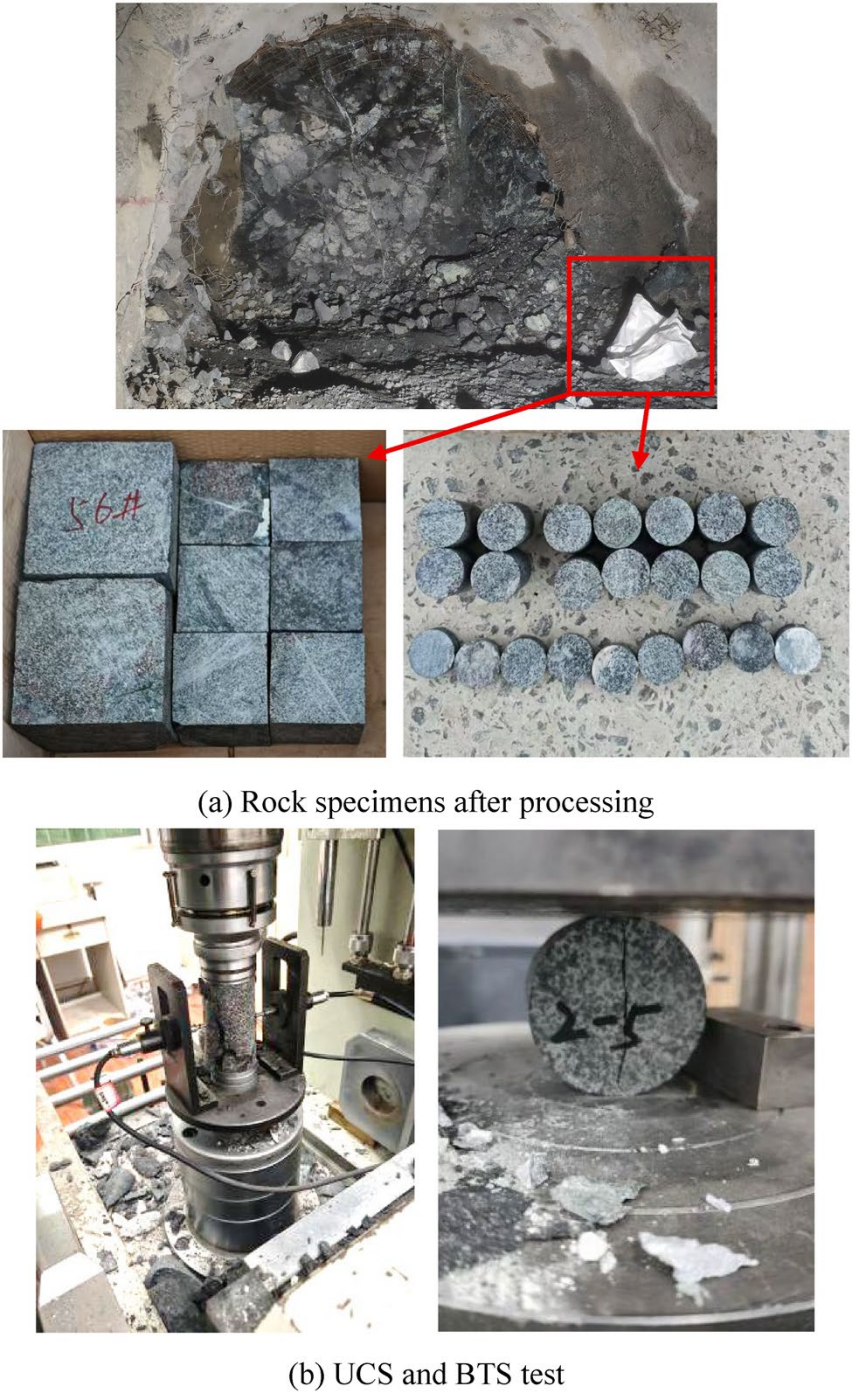
Conventional rock mechanical test of rock specimen 1300a.

Rock specimens were sampled from four mining drifts in the Jinchuan No. 3 Mine, where the primary mineral resources are copper, nickel and cobalt. Conventional rock mechanical tests were conducted, including UCS (uniaxial compression strength) and BTS (Brazilian splitting tensile strength). Three samples were used for each test, and the result represents the average of the three tests. The results indicate that the dry UCS of the ore rock ranges from 125.24 to 181.42 MPa, and the saturated UCS ranges from 104.91 to 150.69 MPa. The tensile strength varies between 7.7 and 12.2 MPa, classifying the rock as a typical hard rock. Table 1 gives the mechanical properties of the hard rock from the Jinchuan No. 3 Mine. This study selects rock specimen 1300a to conduct rock linear cutting tests, as the cube rock specimens presented in Fig. 2a, which is of a UCS of 125 MPa and a BTS of 7.7 MPa.

**Table 1:** Conventional mechanical properties.

Rock specimen	Density *ρ* (g/cm^3^)	Elastic modulus* E* (GPa)	Poisson’s ratio *ν*	Dry UCS *σ*_c_ (MPa)	Saturated UCS *σ*_c_ (MPa)	BTS *σ*_t_ (MPa)
1300a	2.96	24.32	0.19	125.24	104.91	7.66
1300b	3.02	25.61	0.14	144.01	147.25	8.33
1330	3.03	20.83	0.22	181.42	150.69	12.19

Because of the high rock strength, which exceeds the capabilities of most of the mining equipment, traditional drilling and blasting mining methods have been predominantly used at the Jinchuan No. 3 Mine. With the advancements of mining equipment performance over recent decades, however, and to eliminate safety hazards associated with blasting operations while improving the efficiency of single mining drift, the Jinchuan No. 3 Mine began exploring new non-blasting mechanical mining methods in 2024. These methods include conical pick cutting and the disc cutter cutting. For this purpose, rock linear cutting tests were conducted to assess the mechanical properties of rock cutting and evaluate the feasibility of mechanical mining by disc cutter-related mining equipment.

### Rock LCM

**Fig. 3:**
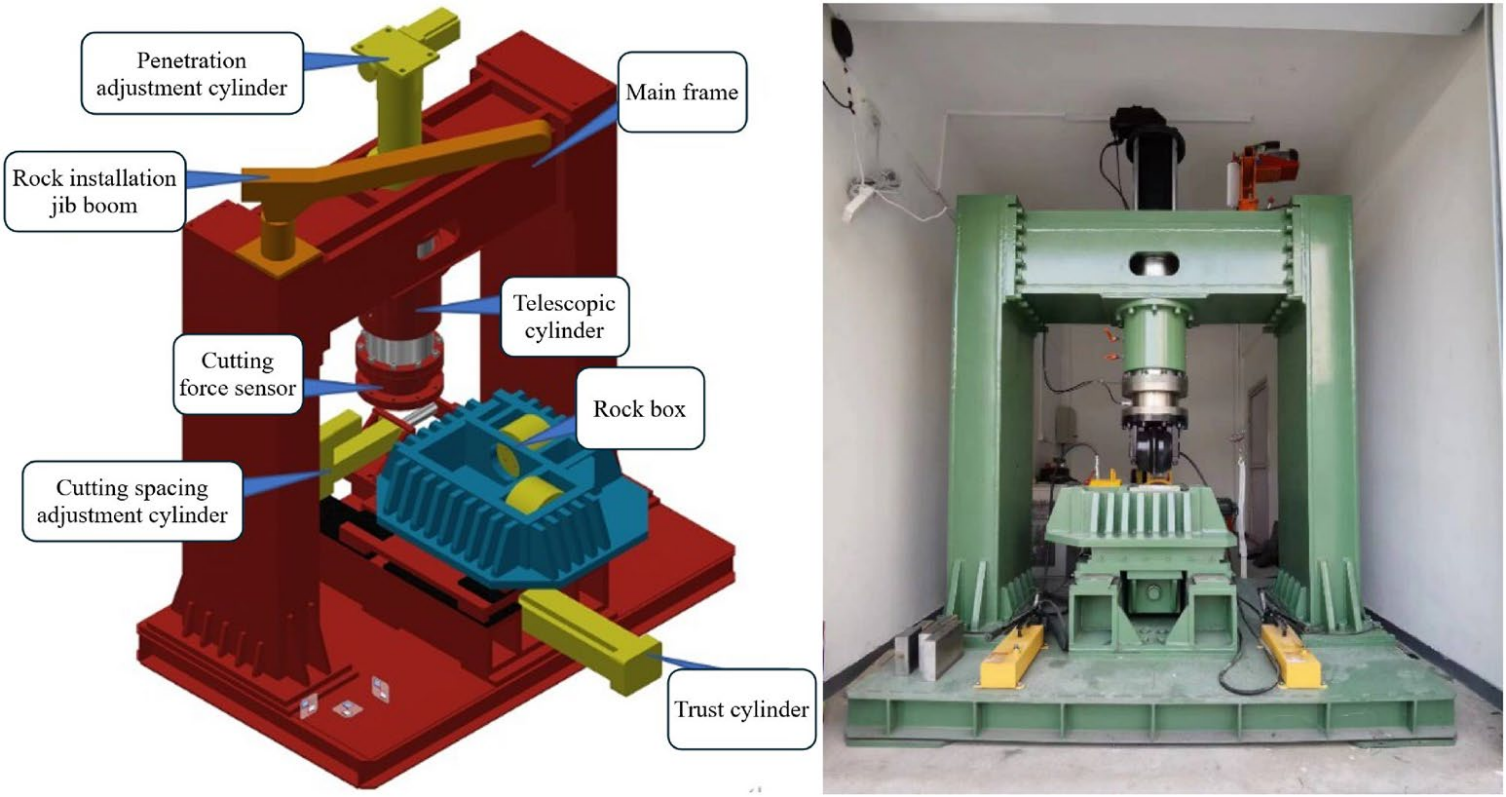
Full scale linear cutting machine.

Rock LCM are classified into two categories in general: small scale^35^ and full scale^36,37^. Small-scale machines offer advantages such as reduced laborer and time requirements, more readily available and inexpensive rock samples. However, these machines have limited stiffness and load capacity^38^. The LCM used in this study is full scale, as presented in Fig. 3. Its main components include the main frame, penetration adjustment system, rock cutting trust system and auxiliary system. In detail, the LCM adopts three high-precision servo electric cylinders to drive the rock box for rock breaking and penetration adjustment. Moreover, a confining pressure loading system is integrated into the rock specimen box, which is composed of manual hydraulic cylinders. For rock specimen measuring 350×200×200 mm, the confining pressure can reach up to 30 MPa. The maximum force applied in the penetration direction is 300 kN, and 150 kN in the rolling direction. The cutting velocity, driven by the trust cylinder, can be adjusted from 0 to 0.3m/s.

The LCM data acquisition system consists of force sensors, a pressure transmitter and a data collection card. It picks up sensor signals, and then force signals are converted to voltage signals, which are collected accurately with a data collection card. The data acquisition system has an accuracy of 0.5% and a sampling rate of 20 kHz. Besides, the data processing system supports exporting test data in multiple file formats, such as EXCEL, MATLAB. Hence, the test data editing, classification and fitting are all available, including for data depth mining and processing.

### Rock linear cutting test

**Fig. 4:**
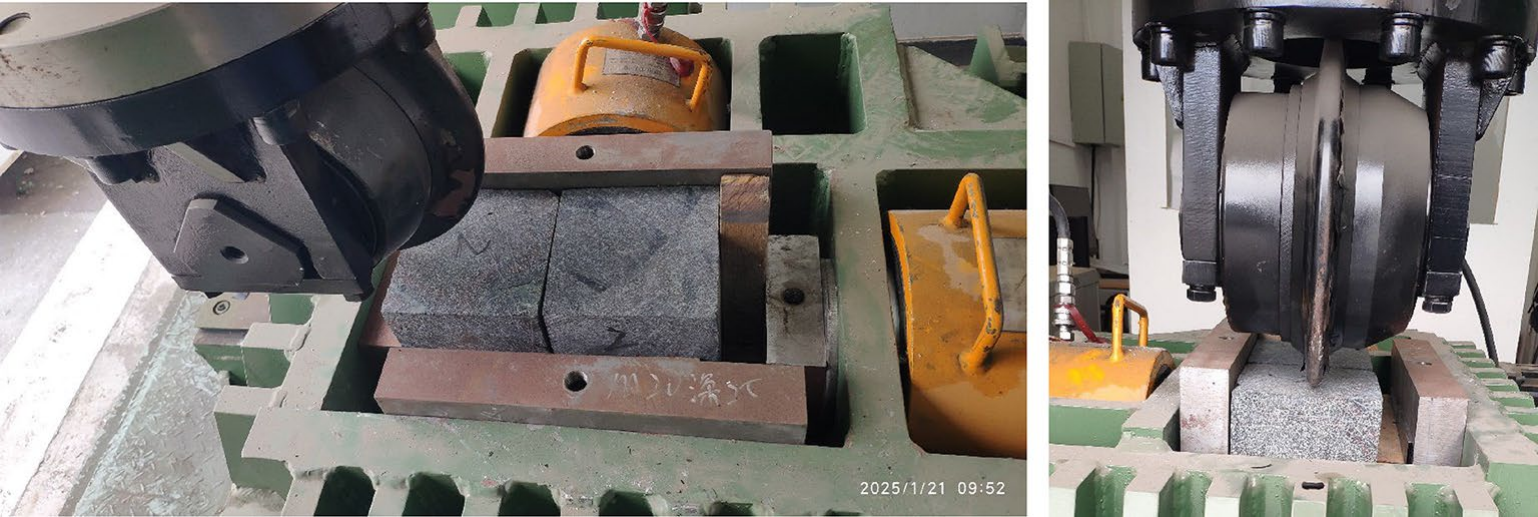
Rock specimen installation in the LCM.

The rock specimens are cubes with a side length of 150 mm. The dis cutter is a 12-inch full-size cutter with a diameter of 305 mm, featuring a flat blade cutter ring, which is a standard cutter ring. The rock specimens are installed as shown in Fig. 4. There are two rock specimens in total, which are used for rock cutting tests under penetration depths of 2 mm and 4 mm, respectively. To ensure the accuracy of the cutter penetration depth, the rock surface is first pre-pressed to achieve a penetration of 0 mm before the formal rock cutting tests. Similarly, under different penetration depth conditions, the position of the rock specimen box is adjusted to ensure an initial cutting displacement of 0 mm. The cutting speed is set at 1 mm/s, and confining pressure is neglected. The cutting grooves are located at the midline of the rock surface. Besides, the accuracy of the pressure and displacement sensors is tested and calibrated by inspection equipment before the tests. During the tests, cutting forces and displacement are recorded in real-time, and force evolution curves versus displacement are plotted via the data acquisition and processing system. After the tests, the rock cutting mechanical properties are analyzed.

It should be noted that the rock specimens in this study are relatively small due to the difficulty of sampling larger rock blocks in mining drifts and processing larger specimens in the laboratory. This limitation will be addressed in subsequent experiments by improving sampling capacity and the performance of the LCM. Also, many factors affect the rock cutting mechanical properties, including the cutting speed, penetration depth, the geometry and size of the disc cutter, and the heterogeneity of rock specimens. Therefore, further research using more rock specimens is necessary. The research on these two specimens provides a calibration basis for subsequent numerical simulation studies.

### Rock cutting mechanical property

**Fig. 5:**
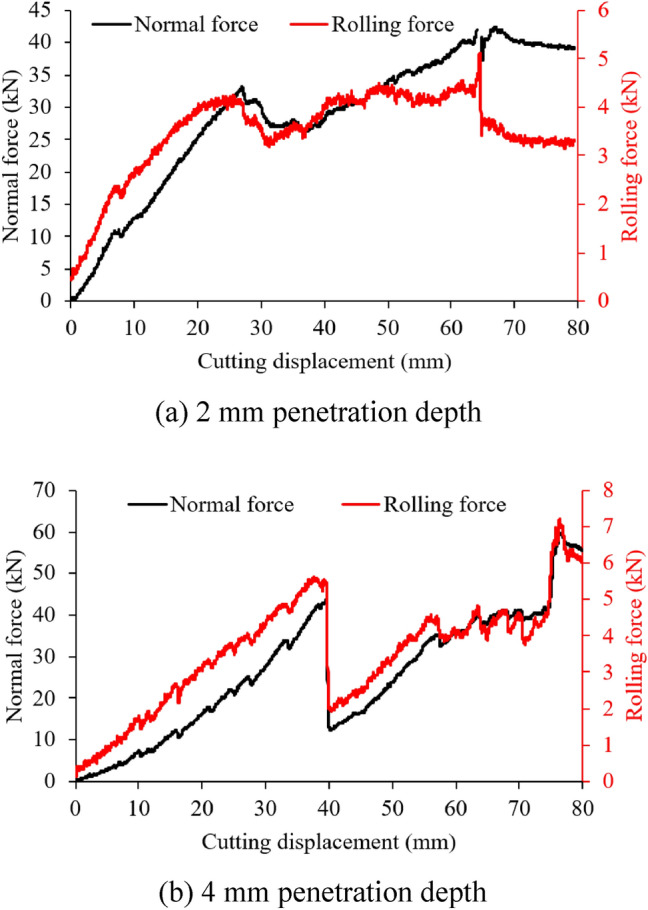
Rock cutting force evolutions obtained from the LCM test.

Cutting force evolutions over displacement under penetration depths of 2 mm and 4 mm are demonstrated in Fig. 5. On the basis of the cutting force curves, the maximum normal forces are about 40 kN at 2 mm penetration and 60 kN at 4 mm penetration. The maximum rolling forces are about 5 kN and 7 kN at 2 mm and 4 mm penetrations, respectively. Accordingly, as the penetration depth of the disc cutter increases, the maximum cutting force also increases. Besides, it can be seen that during the rock breaking process with the disc cutter, the cutting process occurs in a stepwise manner. After comparing the positions where the cutting force drops significantly with the positions where larger volumes of rock chips are produced, it is evident that these positions coincide. This indicates that the generation of rock chips is accompanied by a reduction in cutting force. Moreover, the evolutions of rolling force and normal force remain basically consistent, implying that cutting forces both load and unload in the normal and rolling directions during rock breakage.

**Fig. 6:**
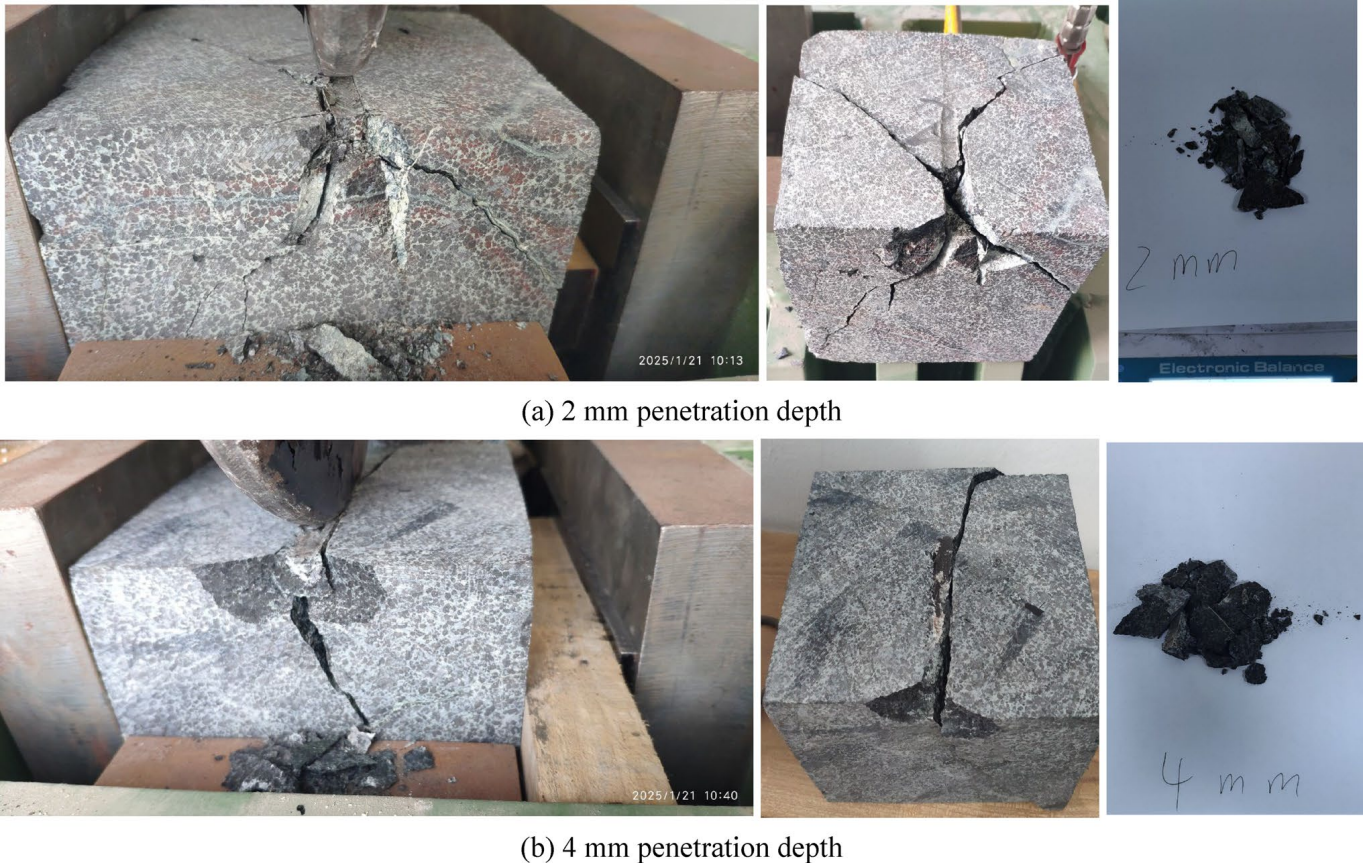
Rock breaking status after disc cutter cutting.

Fig. 6 exhibits the rock breaking patterns under two different penetration depths, clearly showing the macroscopic fracture propagations. When the disc cutter touches the edge of the rock specimen and produces cutting forces, larger volumes of rock breakage initially occur along the edge of the cube rock specimen, distributed on both sides of the disc cutter. As the disc cutter rolls with a constant penetration depth, a crushed zone forms under the contact area between the cutter and the rock surface. Cutting forces are transmitted into the rock interior through the crushed zone, if these forces exceed the rock strength, micro cracks develop. As for macro fractures, two propagation patterns are observed: fractures expand either along the cutting direction or laterally to both sides of the cutting direction. These propagations occur not only on the cutting surface but also in the penetration direction.

After the experiment, the cut rock produced under different penetration conditions was collected and weighted. At the penetration of 2 mm, the cut rock mass is 30.75 g. The rock density is 2.9 g/cm^3^, then the cut rock volume as 10.6 cm^3^. It should be noted that the larger rock chips produced when the disc cutter initially touches and cuts the edge of the cubic rock specimen were not included in this measurement. Similarly, at the penetration depth of 4 mm, the cut rock mass and volume are 27.47 g and 9.5 cm^3^, respectively. The specific energy (SE) can then be calculated by Eq. (1)^39^.

1 $$SE={F}_{\mathrm{r}}l/V$$

Where, *F*_r_ is the mean rolling force, *l* is the cutting displacement and *V* is the cut rock volume.

The mean rolling forces are 3.5 kN and 3.6 kN at 2 mm and 4 mm penetration, respectively. Considering the same cutting displacement of 80 mm, their SEs are 7.33 kWh/m^3^ and 8.42 kWh/m^3^. Due to the limited number of rock specimens tested, the correlation between penetration and SE remains uncertain. Meanwhile, because the rock specimen box of the LCM employed in this study is relatively small, it is unsuitable for research on cutter spacing. Consequently, further numerical simulation researches are necessary.

## Numerical simulation

### Numerical model set-up and calibration

To further study the mechanical properties of hard rock cutting by disc cutters, especially the micro crack development patterns, this study employs PFC3D 7.0 (Particle Flow Code in 3 Dimensions, https://www.itascacg.com/), a particle flow code, to set up a rock cutting model and reproduce the cutting process by the disc cutter. The numerical models of the rock specimens are built using Voronoi block elements rather than spherical particles. The particle shapes of the Voronoi blocks are more irregular and better represent the natural mineral particle shapes found in rocks. These block elements are bonded by contact elements initially, with cracks forming upon bond failure of these contacts. The contact model for the rock specimens adopts the soft-bond model, with the relevant parameters listed in Table 2. The contact model between the rock specimen (block elements) and the disc cutter (wall element) is the linear model.

**Table 2:** Micro-properties of rock specimen.

Property	Parameter
Minimum block geometric size* d* (mm)	5
Block density *ρ* (kg/m^3^)	2970
Contact friction coefficient *μ*	1
Contact effective modulus* E* (GPa)	10
Contact friction angle *φ* (°)	60°
Contact cohesion* c* (MPa)	50
Contact tensile strength *σ*_t_ (MPa)	0.2

**Fig. 7:**
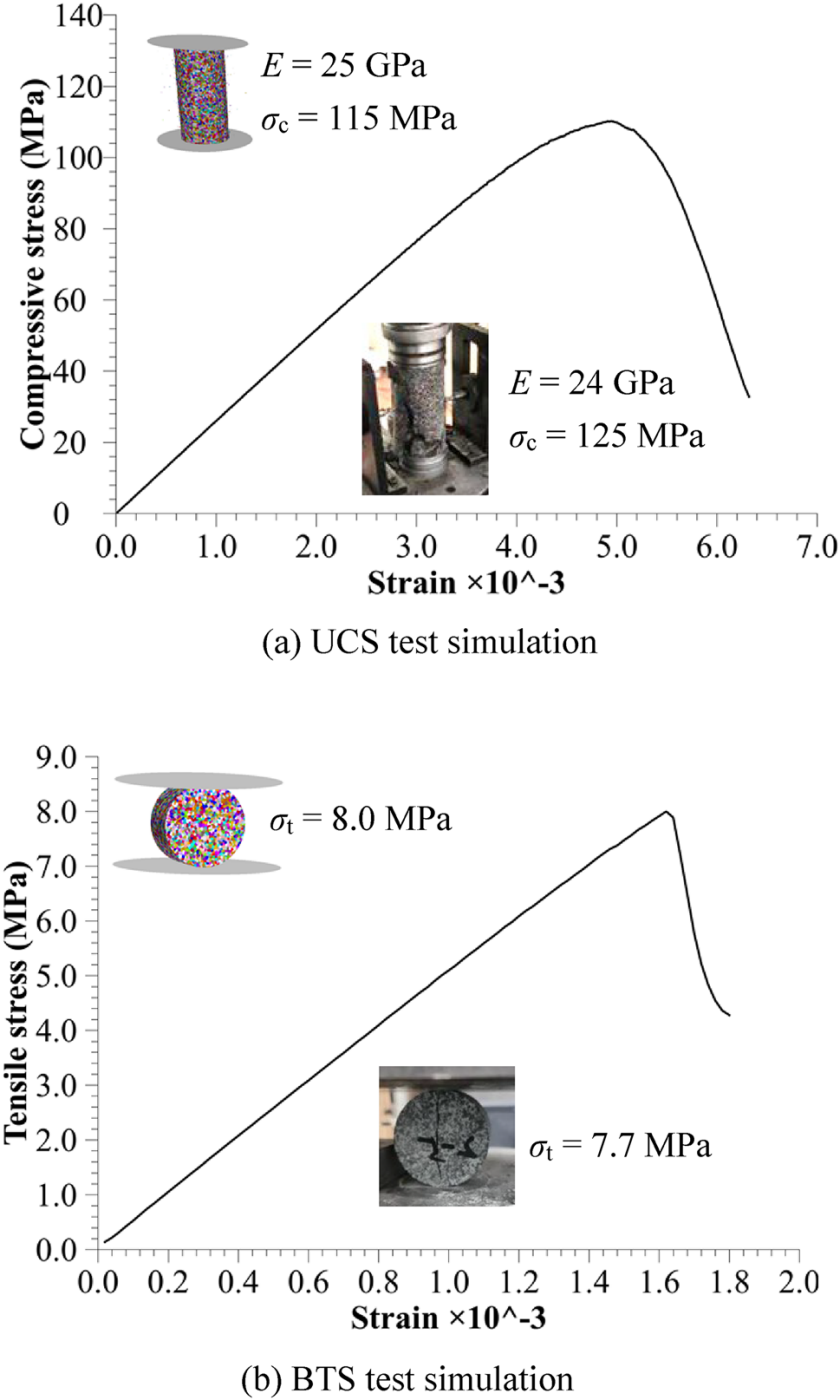
Numerical simulation calibration on mechanical properties of rock specimen.

Before rock cutting simulation, UCS and BTS tests are simulated to validate the numerical model and parameters. Fig. 7 compares laboratory results from conventional rock mechanical tests with simulation results, showing a good consistency in the rock mechanical properties between the two approaches. The errors between simulated and experimental UCS and BTS are about 8% and 4%, respectively. It should be noted that the parameter set mentioned in Table 2 is not unique but represents most suitable set that simultaneously meets the results of both conventional mechanical tests and rock cutting tests. According to the calibration procedure described in the PFC software manual, micro-parameters are calibrated by performing a series of tests on samples with assumed parameters and comparing the results to the desired response of the real intact material. When a match has been found, the corresponding parameter set can be used in the full simulation.

**Fig. 8:**
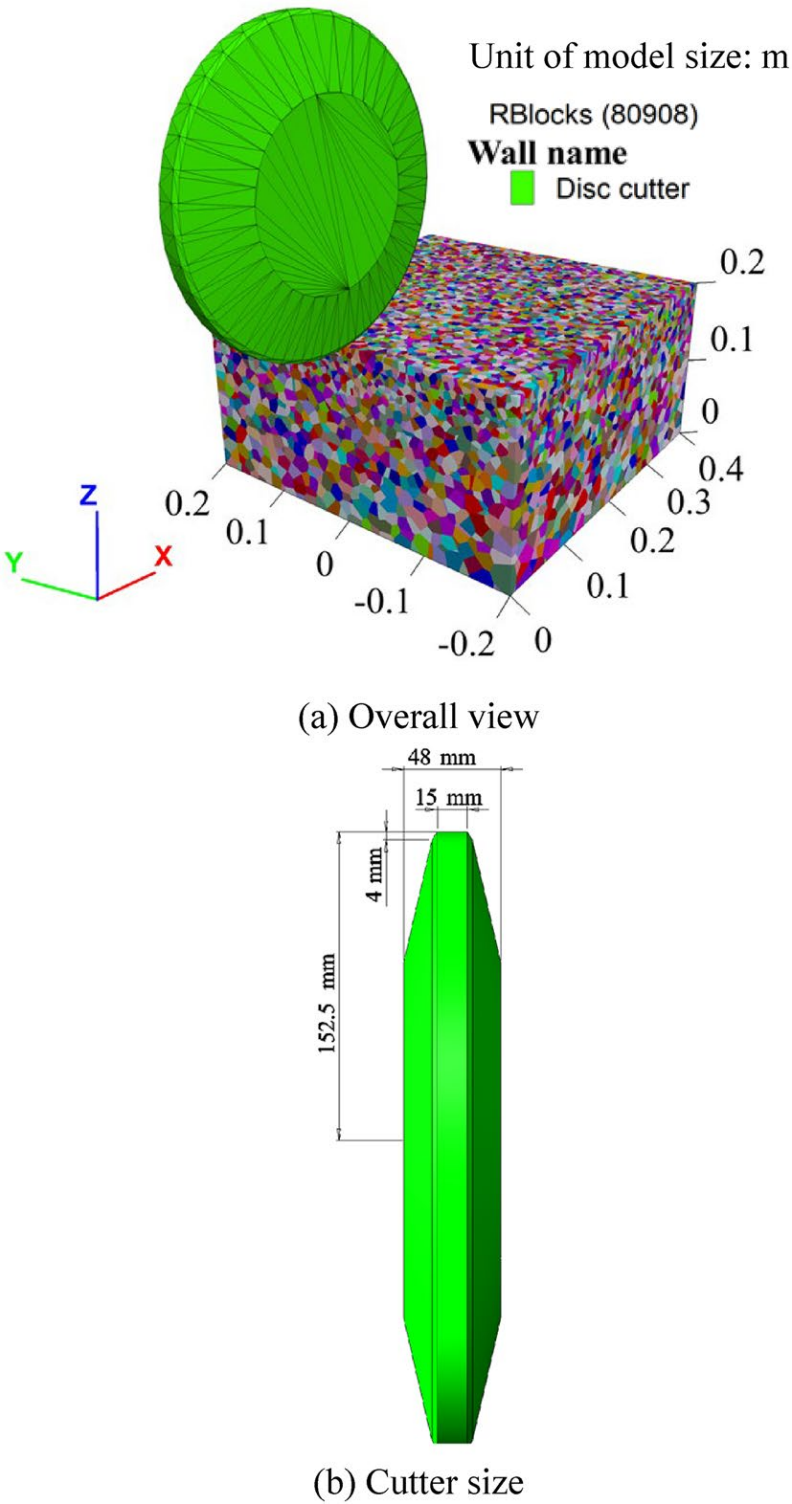
Numerical model of single disc cutter rock cutting.

Subsequently, numerical simulations of rock cutting by the disc cutter are implemented. For the single disc cutter rock cutting simulation, the rock specimen numerical model measures 0.4 × 0.4 × 0.2 m (X×Y×Z), as shown in Fig. 8a. The model consists of 80908 blocks in total, with an average Voronoi block size of about 5 mm in the upper area of the rock specimen. The bottom boundary of the model is fixed, while the four side boundaries are free. The disc cutter has a diameter of 12 inches, matching that used in the LCM test. Similar to the LCM test, the simulation considers two penetrations: 2 mm and 4 mm. The linear cutting velocity along the cutting direction is set to 10 m/s to improve computational efficiency.

To simulate rolling motion, an angular velocity (*ω*) is applied to the disc cutter, calculated by Eq. (2) based on the linear velocity (*v*).

2 $$\omega =2v/d$$

Where, *ω* is the angular velocity, *v* is the linear velocity and *d* is the cutter diameter.

**Fig. 9:**
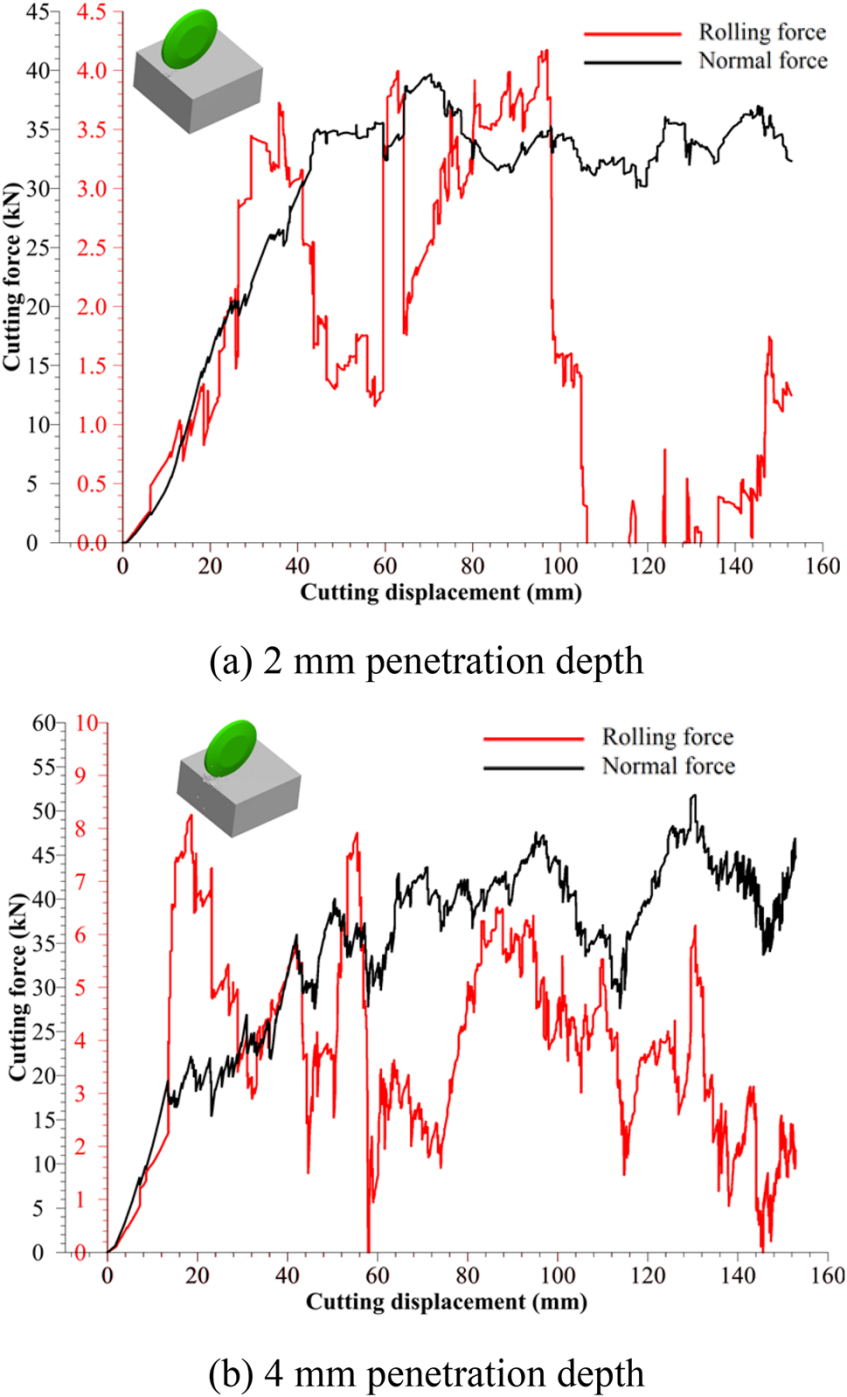
Rock cutting force evolutions obtained from the numerical simulation.

During the rock cutting simulation, the simulated cutting forces are recorded (see Fig. 9) and then compared with those obtained from the LCM tests mentioned in Fig. 5. The simulation shows a maximum normal force of 40 kN and a maximum rolling force of 4 kN at a penetration depth of 2 mm, which aligns well with the LCM test data. Similarly, at a penetration depth of 4 mm, the simulated cutting forces are about 55 kN (normal force) and 8 kN (rolling force), respectively.

To sum up, these results support the reliability of the numerical model and parameters, as the simulated rock mechanical and rock cutting properties are both anastomotic with the experimental tests. Therefore, it is reasonable to conclude that the simulation results accurately reflect the crack propagation characteristics, despite not accounting for potential virgin rock flaws within the rock specimen. Future research could also consider the effect of confining pressure on rock cutting properties.

### Crack propagation characteristic

**Fig. 10:**
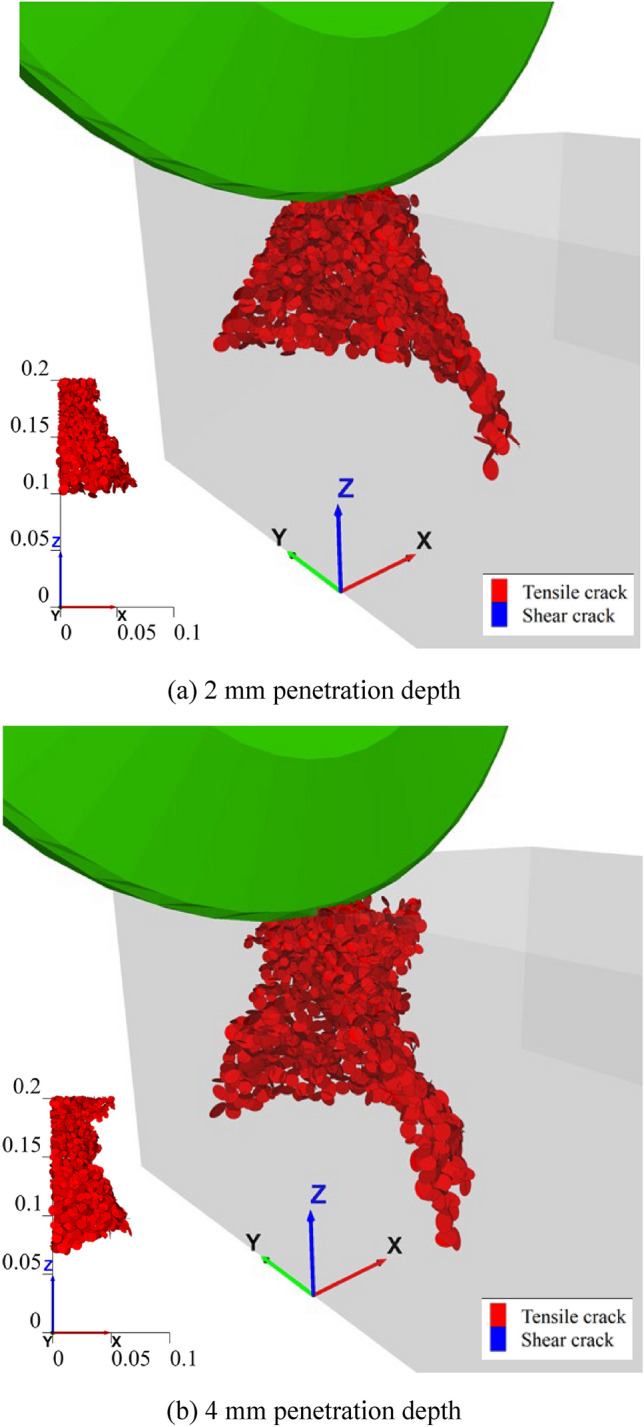
3D spatial characteristics of rock cracks when cutting the edge of the cube specimen.

In PFC3D, the generation and propagation of rock crack are characterized by the failure of bonding contacts between rigid blocks. At the initial stage, when the disc cutter breaks the rock specimen, specifically at the cutting edge of the cube specimen, the 3D spatial characteristics of the rock cracks are shown in Fig. 10. As illustrated, the initial crack under the cutter disc forms a semi-conical shaped surface, consisting entirely of tensile cracks. Fig. 10 also confirms that the depth of crack development increases with penetration depth.

**Fig. 11:**
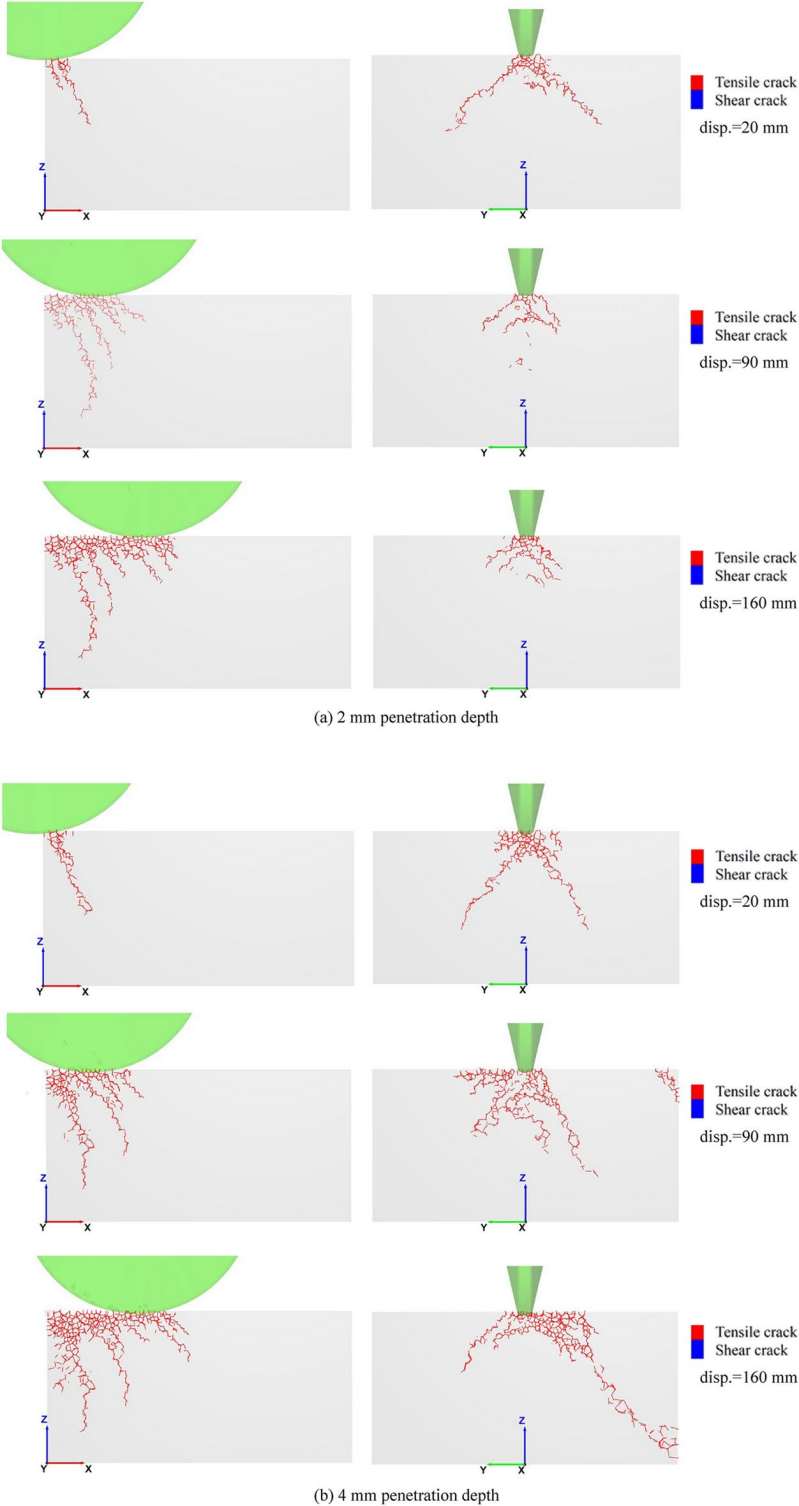
Rock crack characteristic with cutting displacement.

As the cutting displacement (abbr. disp.) goes on, the crack propagation patterns along the rolling direction and cross-section are shown in Fig. 11. It reveals that the rock continuously generates multiple semi-conical shaped cracks, under the rolling and penetration action of the disc cutter. The geometric dimensions of these semi-conical shaped cracks initially decrease at and eventually stabilize. Comparing the crack characteristics at 2 mm and 4 mm penetration depths further emphasizes that greater penetration results in increased depth and width of fracture propagation, facilitating the easier rock breakage and chip stripping.

**Fig. 12:**
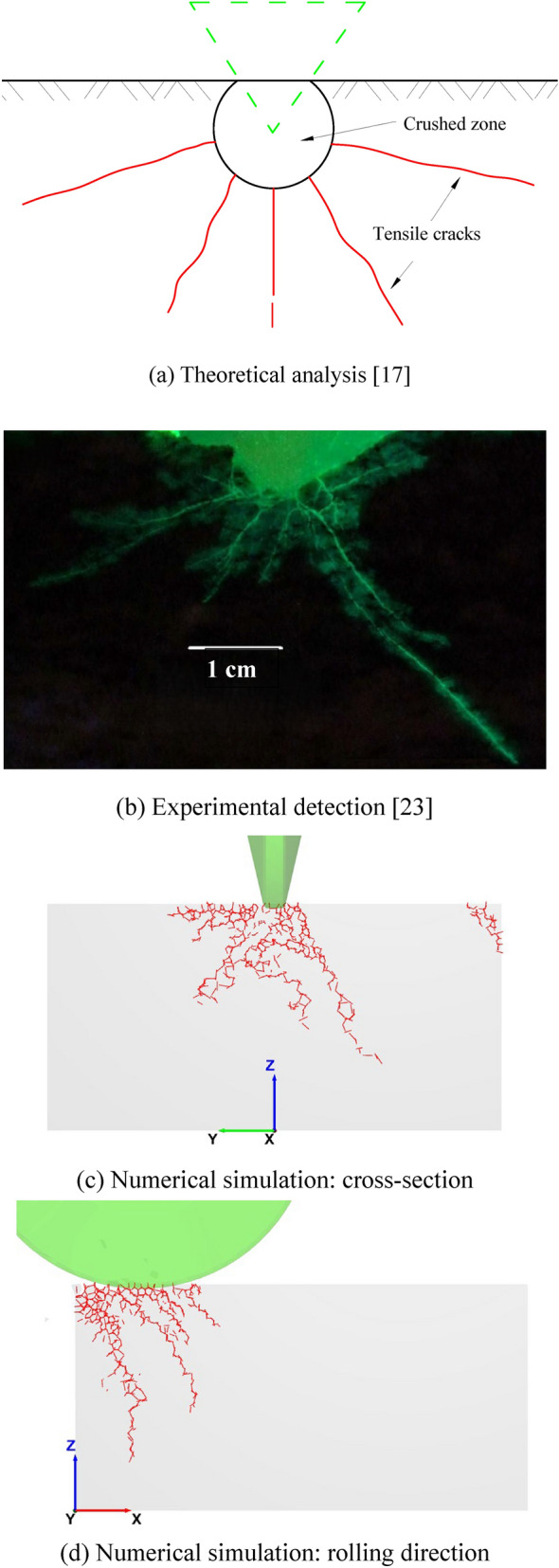
Consistency verification of crack propagation characteristics.

Furthermore, this study compares the crack characteristics obtained from theoretical analysis^17^, experimental detection^23^ and numerical simulation, as exhibited in Fig. 12. The comparison verifies that the numerical simulation results are consistent with those from the other two methods. What’s more, the simulation results highlight that tensile crack dominate the rock cutting process by the disc cutter. Additionally, the investigation of crack patterns along the rolling direction is also feasible using numerical simulation.

### Optimal cutter spacing

**Fig. 13:**
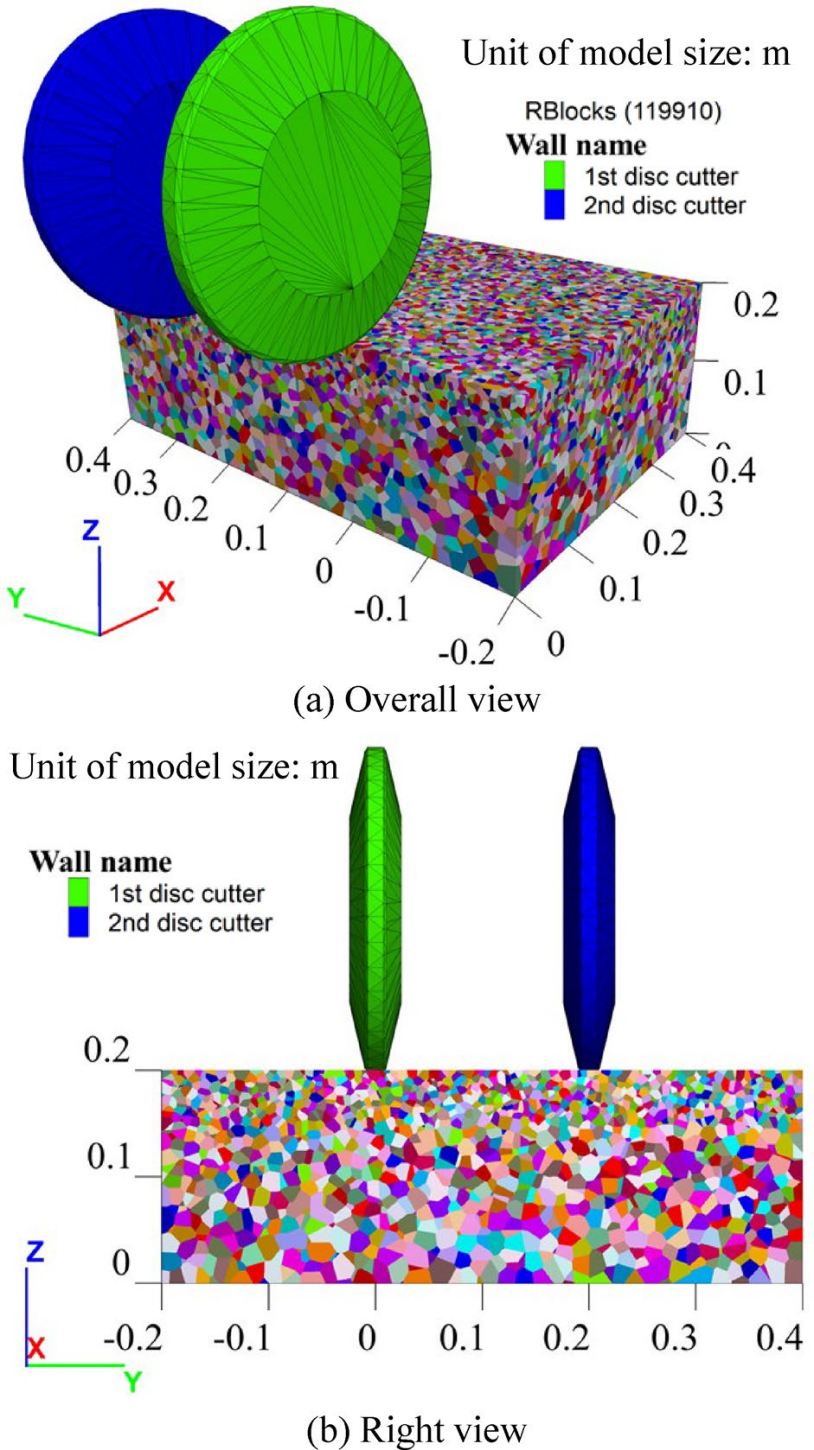
Numerical model of double disc cutters rock cutting.

The cutter head is the core component responsible for breaking rock in cutter-based mining equipment. To optimize the arrangement of disc cutters on the cutter head, it is essential first to study the optimal cutter spacing when using double disc cutters for rock breaking. The optimal cutter spacing must ensure efficient rock chip formation^40^, meaning that the cracks on the cross-section effectively interpenetrate. Hence, the numerical simulations of rock cutting by double cutters were conducted to determine the optimal cutter spacing. The numerical model for double cutters cutting rock is set to 0.4 × 0.6 × 0.2 m (X×Y×Z), as shown in Fig. 13, including 119910 blocks in total. The main model settings and parameters are similar to those used for single cutter cutting, as mentioned in Fig. 8. The penetration depth for cutters is set at 4 mm, with a cutting displacement of 160 mm. The second disc cutter performs rock cutting after the first cutter. Four cutter spacing scenarios were considered: s= 50, 100, 150 and 200 mm.

**Fig. 14:**
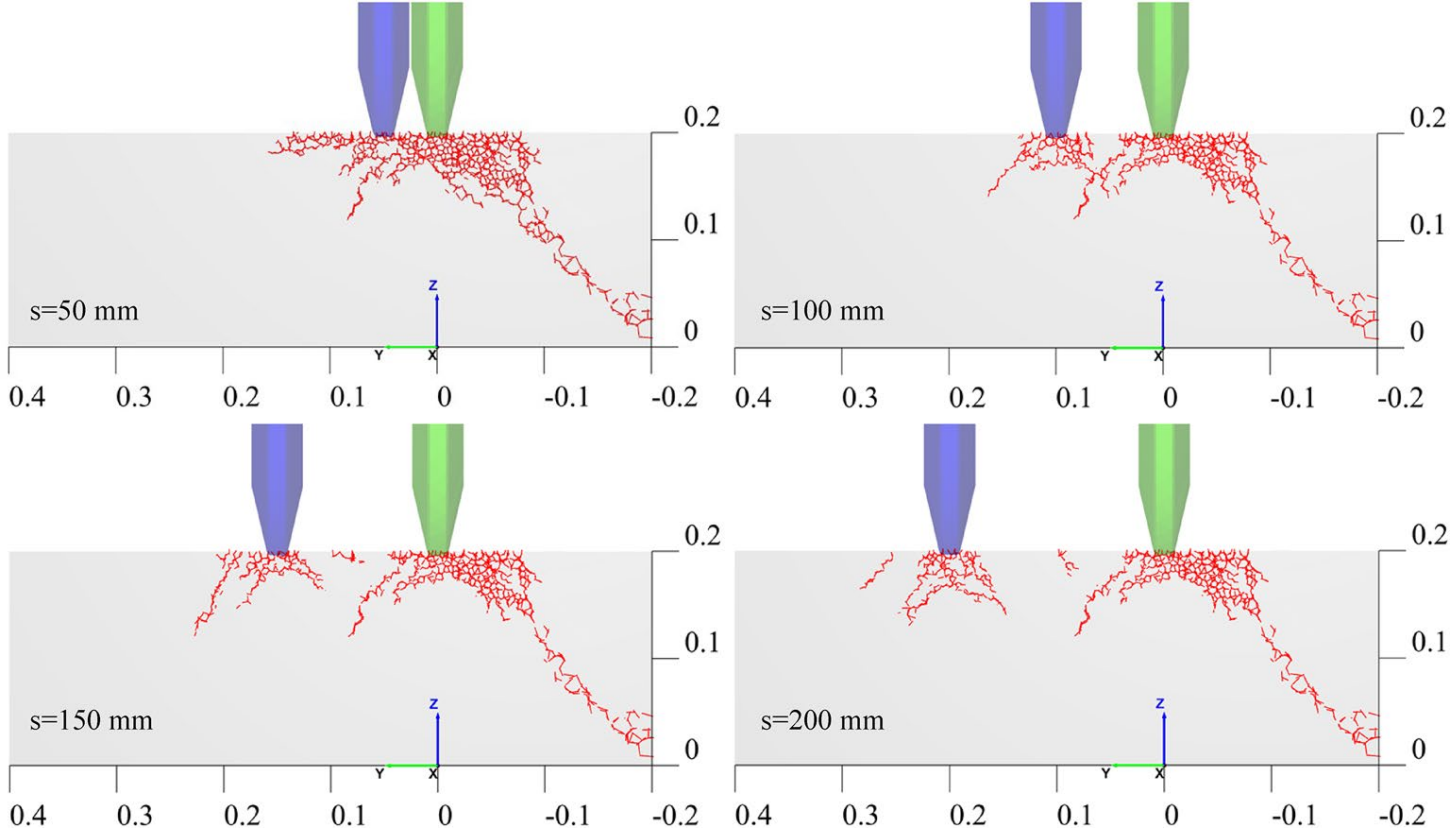
Rock crack characteristic after double disc cutters cutting.

On the basis of the simulation results, the crack developments in the rock specimen induced by the double disc cutters are presented in Fig. 14. It demonstrates an optimal cutter spacing of about 100 mm at a penetration depth of 4 mm when adopting the 12-inch disc cutters to break hard rock in the Jinchuan No. 3 Mine. Larger spacing prevents cracks from fully interpenetrating, while smaller spacing makes denser cracks and more fragmented rock chips. Naturally, the optimal spacing requires further study when changing relevant factors, such as cutter size, cutter shape and penetration depth.

## Feasibility analysis of mechanical mining

### Wheel-type cutter head scheme of hard rock mining equipment

The cutter head is a critical engineering component for TBMs and Mobile Miners, and its design scheme directly affects excavation and mining efficiency, safety, and adaptability to diverse geological conditions. The cutter arrangement schemes of cutter head must balance multiple factors, such as cutter spacing, rotational speed and material durability, to optimize penetration depth while minimizing wear and energy consumption, especially in challenging terrains like hard rock or fault zones. Although extensive research has established the design process for TBM cutter heads, studies on Mobile Miner cutter heads remain relatively scarce.

**Fig. 15:**
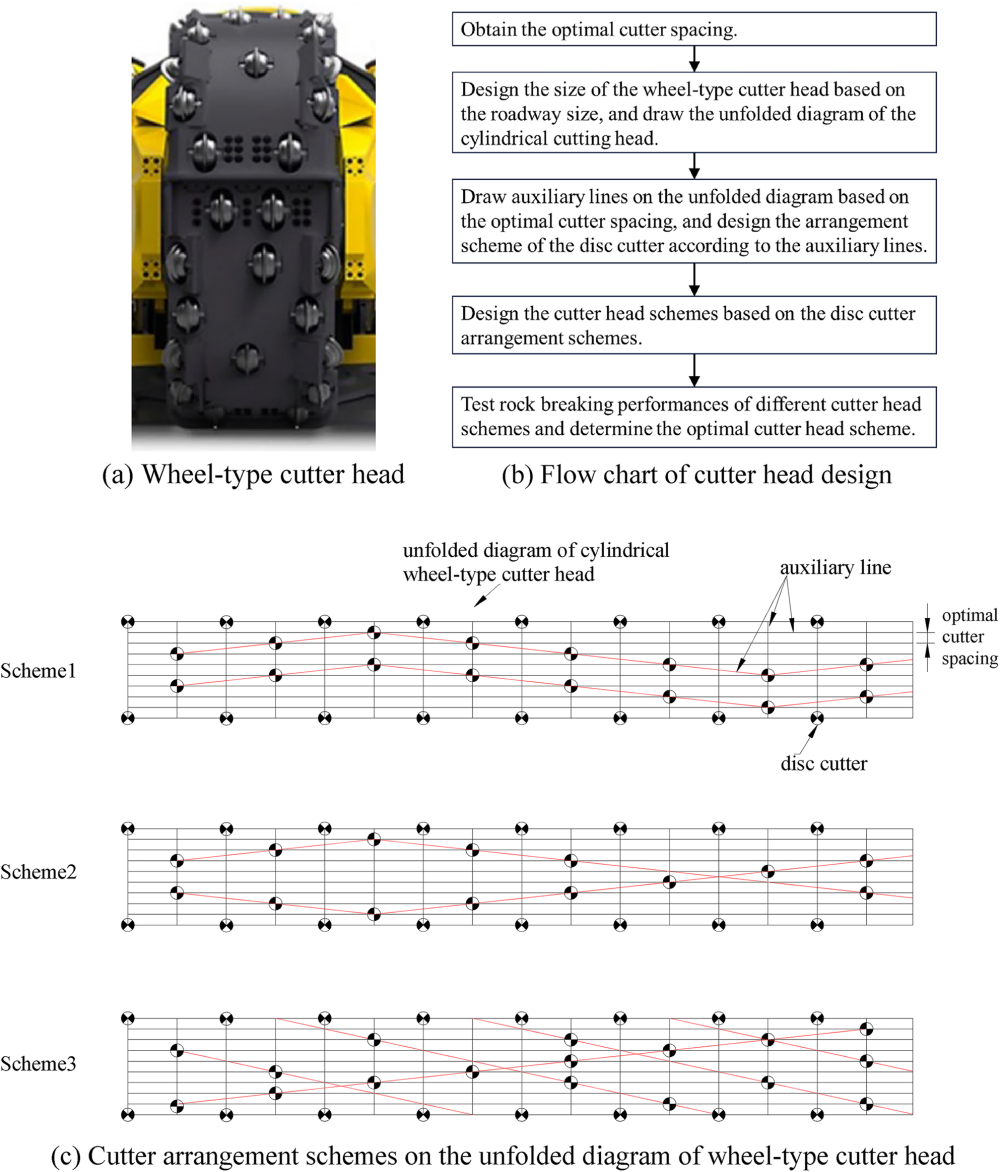
Approach to designing cutter arrangement of wheel-type cutter head.

As an innovative technology, this study proposes a novel approach to designing the cutter arrangements for wheel-type cutter heads. Fig. 15a presents a classical wheel-type cutter head from the Epiroc Mobile Miner series. The design flow chart is shown in Fig. 15b, and Fig. 15c gives three specific cutter layout scheme examples. To protect business secrets, specific scheme parameters are not disclosed, such as cutter size, optimal cutter spacing and penetration.

Specific to the disc cutter arrangement schemes, Scheme 1 is advantageous for maintaining the consistent optimal tool spacing. Scheme 2 features a symmetrical cutter head arrangement, which helps prevent concentrated eccentric loading on one side of the cutter head. In contrast, Scheme 3 has a single set of concentrated disc cutters, which contributes to initial cutting and rock breaking. Hence, Scheme 1 is recommended.

### Mining efficiency estimation and equipment requirements

Most scholars use the field penetration index (FPI) as an evaluation indicator for the construction performance of disc cutter-based excavation equipment^41,42^. However, since FPI is based on TBM projects and not on Mobile Miner projects, this study puts forward a procedure to estimate mining efficiency. The LCM test results confirm penetration index (PI) values of 20 (for 2 mm penetration) and 15 (for 4 mm penetration), with an average PI of 18 for the 12-inch cutter.

Furthermore, when the Jinchuan No. 3 Mine adopts the hard rock mining equipment, Mobile Miner 40V^43^, it is assumed that the disc cutter-based wheel-type cutter head rotates at 5 revolutions per minute, that is, rotational speed (*r*) = 5 r/min. During the rock breaking process, half of the disc cutters are always in contact with the rockmass. This half amount is 16 cutters, and the total number (*N*) is 32. The width of the cutter head is 1.35 m, and the cross-section of the mining drift is 4 × 4 m, requiring three cuts (*n* = 3) to complete the cross-section. If the effective rock-breaking time (*t*) is taken as 12 hours per day, the mining efficiency AR (advance rate) of the hard rock mining equipment can be estimated via Eq. (3).

3 $$AR=\left(60\times p\times r\times t\right)/n$$

Where, *p* is the penetration depth, *r* is the rotational speed of the cutter head, *t* is the effective rock-breaking time, *n* is the number of cuts required for one cross-section.

The required maximum thrust *F*_t_ for the mining equipment is evaluated roughly by Eq. (4).

4 $${F}_{t}=0.5\times N\times {F}_{n}$$

Where, *N* is the number of disc cutters on the wheel-type cutter head, *F*_n_ is the maximum normal force exerted by a single disc cutter.

When taking a penetration of 2 mm, the mining efficiency AR is approximately (60×0.002×5×12)/3 = 2.4 m/d, and the maximum thrust required by the cutter head is approximately 16×40 = 640 kN. Similarly, for a penetration of 4 mm, AR is about (60×0.004×5×12)/3 = 4.8 m/d, and the required maximum thrust is about 16×60 = 960 kN.

As penetration increases, the predicted results for mining efficiency AR and the required maximum thrust *F*_t_ are summarized in Table 3, where the peak normal force of the disc cutter is calculated as *p*×PI. Accordingly, hard rock mining equipment will achieve a higher advance rate in the Jinchuan No. 3 Mine by increasing the penetration of the disc cutter.

**Table 3:** Estimation of mining efficiency and equipment requirements.

Penetration *p* (mm)	Mining efficiency AR (m/d)	Required maximum thrust *F*_t_ (kN)
2	2.4	640
4	4.8	960
6	7.2	1728
8	9.6	2304
10	12.0	2880

### Mechanical mining method for hard ore rock

**Fig. 16:**
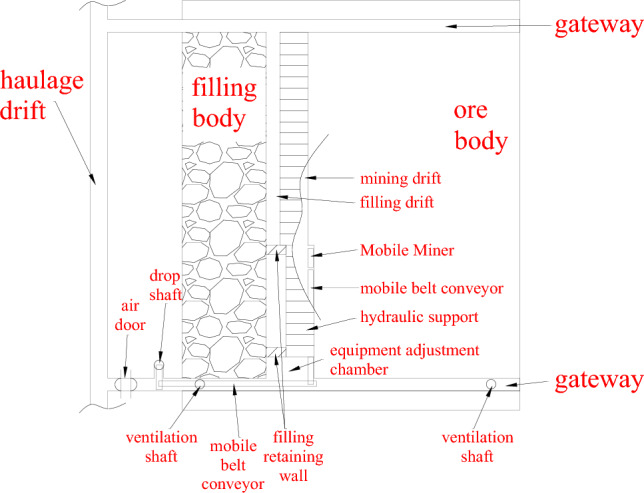
Mechanical mining method for the mines with hard rock based on the Mobile Miner.

In addition to innovations in equipment performance, the method of equipment usage also significantly influences tunneling and mining efficiency. Unlike TBMs, which are primarily used for long-distance excavation of a single tunnel or roadway, a Mobile Miner must consider frequent movement within a specific mining area. Referring to the long wall mining method, which is well-established applicable to coal mines^44^, this study proposes a mechanical mining method in metal and non-metal mines. Fig. 16 presents the layout of a mining district suitable for mines with large volumes of hard ore.

Two gateways are excavated parallel to the haulage drift, both perpendicular to the ore body, followed by excavation of the mining drift between the gateways. An equipment adjustment chamber is constructed on the side of the mining drift close to the ventilation shaft. Filling hydraulic supports are then arranged in the mining drift, with the Mobile Miner and mobile belt conveyors positioned under these supports. The mining process proceeds as follows: the Mobile Miner enters the equipment chamber from the gateway and excavates the ore body in parallel to the mining drift. The mined ores are transported to the rear of the hard rock mining machine and then further transported to the drop shaft by the mobile belt conveyors located in the mining drift and gateway. As the hard rock mining machine excavates in the mining drift, the filling hydraulic supports move forward accordingly. Upon completion of mining one drift, the hard rock mining machine and the mobile belt conveyor are withdrawn to the equipment chamber along the mining drift. Meanwhile, filling retaining walls are constructed in the filling drift behind the filling hydraulic supports to complete the filling operation. When mining activities involve potential geological risks, such as rockmass joint development and groundwater factors^12–14^, precautionary measures like pre-grouting and displacement monitoring should be implemented.

## Conclusions

This study investigates the mechanical properties of rock cutting by disc cutters in hard rock and evaluates the feasibility of mechanical mining in a hard rock mine. The main results and findings of this paper are as follows:

(1) The rock mechanical tests indicate that the UCS of the ore rock in the Jinchuan No. 3 Mine ranges from about 125 to 180 MPa, characterizing it as hard rock. The LCM tests reveal the mechanical properties of rock cutting by a single disc cutter, including penetration depths of 2 mm and 4 mm. The UCS, BTS and cutting force values are used to calibrate the numerical model of rock cutting by the disc cutter and its parameters. The numerical simulation results are consistent with the experimental data.

(2) Based on the calibrated numerical model, crack propagation induced by rock cutting is investigated, both along the rolling direction and across the cross-section. The cracks are shown to tension-dominated, and their characteristics have a good agreement with theoretical analysis and experimental observations reported by other researchers. It is also found that the crack shape under the cutter disc forms a semi-conical shaped surface, and the rock continuously generates multiple semi-conical shaped cracks with increasing cutting displacement, with their sizes initially decreasing before stabilizing.

(3) The optimal cutter spacing should ensure the connection between cracks induced by the double-disc cutters. Simulation results suggest a suitable spacing of about 100 mm at a penetration depth of 4 mm for the 12-inch cutter. A larger spacing prevents the cracks from fully intersecting, while a smaller spacing results in denser cracks and more fragmented rock chips. The determined optimal cutter spacing can be used to design the disc cutter arrangement for mining equipment.

(4) This paper proposes a design approach for the cutter arrangements on a wheel-type cutter head, on the basis of the optimal cutter spacing. After evaluating the advantages and disadvantages of three cutter arrangement schemes, it recommends a specific wheel-type cutter head configuration. Furthermore, the paper discusses mining efficiency estimation, equipment requirements and mechanical mining methods for hard ore rock. Overall, mechanical mining using hard rock mining machines is feasible for mines with large volumes of hard ore.

(5) Future work should focus on linear cutting tests using larger volumes of rock specimens, analyzing the effects of confining pressure, penetration depth and cutter shape, conducting parameter sensitivity studies, as well as performing multi-field coupling simulations and field application tests.

## Data Availability

The datasets generated during the current study are available from the corresponding author on reasonable request.
